# Diversity and specificity of lipid patterns in basal soil food web resources

**DOI:** 10.1371/journal.pone.0221102

**Published:** 2019-08-20

**Authors:** Jakob Kühn, Kathlin Schweitzer, Liliane Ruess

**Affiliations:** 1 Institute of Biology, Ecology Group, Humboldt-Universität zu Berlin, Berlin, Germany; 2 Institute of Agricultural and Horticultural Sciences, Department of Crop and Animal Sciences, Division of Plant Nutrition and Fertilisation, Humboldt-Universität zu Berlin, Berlin, Germany; Universidade do Porto, Faculdade de Farmácia, PORTUGAL

## Abstract

Soil food webs are important drivers for key ecological functions in terrestrial systems such as carbon and nutrient cycling. However, soil food web models generally lack quantitative data, mainly due to the shortage in high-throughput methods to describe energy flows. In marine environments, multivariate optimization models (Quantitative Fatty Acid Signature Analysis) and Bayesian approaches (source-tracking algorithm) were established to predict the proportion of predator diets using lipids as tracers. A premise for the application of such models to soil systems is to acquire the fatty acid pattern of a broad range of resources and to reveal potential overlap in their signatures. We present a comprehensive comparison of lipid pattern across widespread taxa of plants (leaves and roots, n = 48), algae (n = 59), fungi (n = 60), and bacteria (n = 62) as basal food web resources. Lipid profiles from microorganisms and algae were assessed from laboratory cultures, whereas plant tissue was derived from an arable field. A lipid library was constructed and multivariate data analyses (hierarchical clustering, nMDS) was used to assess the extent of separation in lipid pattern by species or resource type. The performance of the lipid library was tested by leave-one-prey-out (LOPO) analysis, giving the distinctiveness of the resource (prey) groups. Fungi and plant leaves were correctly assigned based on their lipid pattern with more than 98%, while plant roots and bacteria achieved 88 and 85%, respectively. However, algae were only correctly classified by 60%, pointing to a bias in the herbivore food chain. Fatty acids most important for separation of algae and plant leaves were of the omega 3 type, i.e. 16:3ω3 and 18:3ω3. In plant roots 18:1ω9 was most important, whereas bacteria were distinguished predominantly by methyl-branched fatty acids. Overall, the lipid pattern of major soil food web resources are sufficiently differentiated to allow for qualitative (biomarker) analyses as well as quantitative modelling, yet with precaution in the case of algae.

## Introduction

Fatty acids constitute one of the major cell components, forming the membrane as phospholipids and representing the main energy storage as neutral lipids [1]. Despite this ubiquitous nature, the frequency, diversity and specificity of fatty acids differs among organismic groups. This made them a common choice in chemotaxonomic and food web research, where biochemical methods such as stable isotopes have long become standard [2]. Especially advanced is the use of fatty acids as trophic biomarkers in marine systems, where ω3 polyunsaturated fatty acids (PUFAs) are almost exclusively synthesised by planktonic algae at the food web base. This allows following the trophic transfer of ω3 PUFAs in many invertebrates and vertebrates of the marine food chain [3, 4, 5].

Lipid analysis as a tool to assess trophic interactions in cryptic soil food webs commonly uses a wider array of fatty acids markers [6]. These comprise absolute makers, synthesized via the lipid metabolism of the prey (resource) but not by the consumer, and relative markers, which the consumer is able to synthesize, but that are additionally highly accumulated from the diet [7]. Typical absolute markers are branched-chain (iso, anteiso) and cyclopropane (cy) fatty acids in bacterial resources [8] as well as ω3 PUFAs derived from feeding terrestrial micro-algae [9]. Plant and fungal resources are indicated by the relative markers oleic acid (18:1ω9) and linoleic acid (18:2ω6,9), respectively [6].

Since the establishment as biomarkers in soil ecology, fatty acids have been widely applied to investigate trophic links in the detrital food chain, i.e. consumers of bacteria and fungi, and their predators [10], as well as in the herbivore food chain, i.e. the dietary routing of ω3 PUFAs [9]. Such studies usually focused on a handful of resources/prey and consumers, and only recently lipid analyses were extended to entire consumer communities [11]. While very informative, these studies are largely qualitative in nature, giving little information about the absolute quantities of trophic lipid flow. For this, other biochemical methods such as stable isotope labelling are used [12], employing both bulk element and biomolecule signals. Still, the challenge is to trace fatty acid transfer to higher trophic levels in natural, non-manipulated soil food webs quantitatively.

About a decade ago, a model to infer a consumer’s diet by its specific fatty acid signature, the so-called quantitative fatty acid signature analysis (QFASA) [13], has been established for marine ecosystems. This model tries to minimize the distance between the fatty acid signature composed of resource signatures and the measured consumer signature, with a distance of ‘0’ indicating a perfect fit. As the estimation of a consumer diet is based on the combination of resource signatures, knowledge in the lipid pattern of a wide range of resources–a lipid library–is essential. Such a library is comprised of different resources depending on ecosystem, habitat and the consumer in focus. The QFASA model has been conducted on e.g. seabirds [5], polar bears [4], sea lions [14] and several type of fish [15, 16]. As such, the primary groups in the underlying libraries are zooplankton, phytoplankton and small fish. Therefore it is necessary to construct a fitting library for soil ecosystems, as the prime resources are vastly different.

Still, consumer-resource relationships in soil food webs are poorly understood. Comprehensive diet estimates are particularly difficult due the fact that most faunal decomposers appear to be food generalists rather than specialists [17]. Further, when consuming dead organic matter, meso- and macro-faunal decomposers ingest not only plant tissue but also the adhering micro-consortium of bacteria, fungi, algae, protozoa and nematodes. Nevertheless, three distinct soil carbon and energy channels can be distinguished, the root, bacterial and fungal pathway [18], and trophic fatty acid biomarkers are at hand for their basal resources [6]. However, these resources are usually very diverse in taxonomical, biochemical and structural characteristics. This applies to both phylogenetic differences in lipid metabolism, e.g. *de novo* synthesis of ω3 PUFAs predominantly by algae and higher fungi [19] as well as differences of dominant fatty acids between tissues, e.g. the ratio of oleic to linoleic acid in shoots and roots [20].

Given the scarcity of *a priori* diet data for soil food webs, there is need for a comprehensive lipid library, encompassing a wide range of lipid profiles of major basal resources. This study aims to provide such an overview by sampling, extracting and analysing a large number of lipid profiles in algae, bacteria, fungi and plants. We constructed a lipid library based on a homogenous dataset comprising 50 different resources and in total 229 samples. This lipid library was used to: *i)* assess the fatty acid diversity and variability in basal soil food web resources, *ii)* determine the fatty acid signature specificity and the main fatty acids responsible for that, and *iii)* assign the distinctiveness of resource groups as well as identify confounding groups.

## Methods

### 2.1 Organisms

A lipid library was constructed comprising the four major basal food web resources in soil (plants, algae, fungi, bacteria), which consists of both prior collected fatty acid data as well as newly extracted lipid pattern. Fungal data taken from own experimental data [21, 10] are composed of 60 entries belonging to Basidiomycota, Ascomycota, Mucoromycota (former Zygomycota) and Deuteromycota (for species list see S1 Table). Fungi from the 2002 dataset [21] were sampled from two forests in the UK and cultivated on Pachlewska or malt extract broth at 15°C in darkness, fungi from the 2004 dataset [10] were long term laboratory cultures on Pachlewska or potato dextrose broth, cultivated at 15°C in darkness. The lipid extraction was performed with the same protocol as in the current study (see below chapter 2.2).

Plant data were collected from common agricultural crops, sampled at the “Field demonstration of crop cultivars” (13.295819 E, 52.467956 N), which has been running at the experimental station of crop sciences at the Thaer-Institute, Humboldt-Universität zu Berlin (Germany), since 2006. The climate at the experimental site is classified as semi-continental [22] with an average annual air temperature of 9.9°C and an annual precipitation of 562 mm (standard period 1981–2010). The soil is an Albic Luvisol [23] developed on quarternary deposits. The upper soil horizon (0–30 cm) is described by a soil texture of loamy sand, a low SOC content of < 10 g kg^−1^, and a pH value of 5.8. Plants were grown in an 8-yr crop rotation, which in 2017 was represented by 16 crop species each with 3 to 24 cultivars without replication, of which 8 species, with 3 different cultivars each, were chosen. These crops were *Beta vulgaris*, *Brassica napa*, *Helianthus annuus*, *Lupinus angustifolius*, *Solanum tuberosum*, *Triticum aestivum*, *Triticum durum*, and *Zea mays*; the specific cultivars are given in S1 Table.

The agronomic management represents the conventional local farming and has been constant since 2006: Average mineral fertilisation N:P:K was 81:21:123 kg ha^−1^ yr^−1^, farmyard manure was applied in two of eight years during the first rotation cycle from 2006–2014, liming was performed as required for optimal pH of 5.8–6.3, and chemical plant protection based on disease scoring (see S2 Table for further details). Sampling was performed on the 8^th^ of June 2017 and comprised both root and leaf samples from each of the eight crops. From each plant species, three individual plants (one per cultivar) were chosen randomly, dug out to the ploughing depth of 28 cm, and separated into root and shoot tissue. This resulted in a total amount of 48 samples, i.e. 3 root and 3 shoot samples for each of the 8 crops. Roots were cleaned from earth and both plant parts were stored at −20°C until lipid extraction.

Moreover, 59 samples from 10 species of green algae common in soils and biological crusts were incorporated into the lipid library (for species list see S1 Table). The algae cultures were provided by the SAG Göttingen and maintained on Bold’s basal medium with added vitamins (BBM+V) in a climate chamber featuring 16 hours of light (30 μmol m^−2^ s^−1^) and 8 hours of darkness, air humidity of 50% and a temperature of 15°C for 2 weeks to gain sufficient biomass. Algal samples were stored at −20°C until lipid extraction.

Additionally, 62 samples from 16 strains of bacteria commonly found in soil ecosystems were incorporated into the library, comprising actinobacteria, acidobacteria and firmicutes (for species list see S1 Table). Actinobacteria were cultured on yeast-malt-extract agar for long-term cultivation and grown on standard liquid broth media for mass cultivation for lipid extraction. Acidobacteria were grown on PSY-Agar medium for long-term cultivation and PSY-Liquid broth medium for lipid extraction [24].Acidobacteria cultures were provided by Minnä Mannistö (Luke, Rovaniemi, Finland), while Actinobacteria cultures were provided by Mikka Tarkka (UFZ Halle, Germany). The other bacteria were given by the microbial physiology (Marc Erhardt) and microbiology (Thomas Eitinger) groups from the Humboldt-Universität zu Berlin (Germany). Cultivation at 15°C took between 1 day and 1 week, depending on growth rate and medium. Bacterial samples were stored at −20°C until lipid extraction.

### 2.2 Fatty acids

The established lipid library consists of fatty acid methyl ester (FAME) data. FAMEs were extracted from organisms and tissues via the MIDI protocol (MIDI Inc., Newark, Del.), a simple and fast approach extracting the total lipid fatty acids (TLFAs) of the sample. MIDI consists of four steps, beginning with a hydrolisation and saponification step at 100°C using 1 ml of a solution of 45 g sodium hydroxide, 150 ml methanol and 150 ml ultra pure water. The fatty acids are then methylated with an acidic solution consisting of 325 ml 6.0 N hydrochloric acid and 275 ml methanol, adding 2 ml to each sample. The saponified and methylated FAMEs are extracted via two-phase separation by adding 1.25 ml of a solution of 200 ml hexane and 200 ml methyl-tertiary-butyl ether. Thereafter, the separated organic phase is washed with 3ml of a solution of 10.8 g sodium hydroxide and 900 ml ultra pure water [21]. By this protocol all lipid fractions are gained, i.e. phospholipids, neutral lipids, and glycolipids, representing the entire fatty acids a consumer ingests with the respective biotic resource. Extracted FAMEs were stored at −20°C until analysis.

The quantification and identification of FAMEs was conducted using an Agilent 7890A gas chromatograph and flame ionisation detector with an HP Ultra 2 capillary column (25 m length, 0.2 mm inner diameter, film thickness = 0.33 μm), an injection volume of 2 μl and hydrogen as carrier gas. The temperature program started at 50°C, increasing by 25°C min^−1^ up to 175°C, followed by 3°C min^−1^ up to 230°C (held for 5.7 min). Identification of TLFAs was conducted via the Sherlock Pattern Recognition Software (MIDI) by comparison of sample retention times with a standard mixture.

A second validation step was conducted with an Agilent Series 7890A gas chromatograph coupled to a mass selective detector (Agilent 7000 Triplequadrupole), equipped with a HP5MS capillary column (30 m lenth, 25 mm inner diameter, film thickness 0.25 μm), operating in splitless mode. Injection volume was 1 μl and helium was used as carrier gas. The oven temperature program began at 40°C and increased up to 200°C by 46°C min^−1^, followed by 5°C min^−1^ to 238°C, 120°C min^−1^ to 300°C, held for 2 min. The transfer line temperature was 280°C and a mass range of 40–400 m/z monitored in scan mode.

In addition to the validation of identified fatty acids, e.g. chain-length and number of double bonds, the ion pattern gained by MS was used to distinguish fatty acids, which co-eluted in the FID analysis. This attributed to the ω3 fatty acids typically observed in plant and algae samples, where the separation of the co-eluting 18:1ω9/18:3ω3,6,9, and 16:1ω7/16:3ω3.6,9 chromatographic peaks was conducted. The couple 16:1ω7/16:3ω3,6,9 was separated by measuring the peak area of the two respective fatty acids in the MS total ion count (TIC) and applying the thereby derived proportions to divide their co-elution. Further, 18:1ω9/18:3ω3 were disentangled by measurement of the ion count of ions specific for the fatty acid (m/z 173 in 18:3ω3 and m/z 180 in 18:1ω9) in the MS. Respective amounts were then calculated using a FAME standard with both fatty acids present in known quantities.

### 2.3 Statistics

After acquisition of the lipid pattern from the different organisms, all fatty acids, which were not present with more than 1% in at least one of the samples, were discarded from further analysis. This resulted in a lipid library consisting of 59 fatty acids for specification of 50 different resources, represented by 229 samples. An nMDS with the entire lipid library grouped by the resource (plants, algae, fungi, bacteria) was conducted with all fatty acids as variables. The vegan package in R was used to assess the dissimilarity and the driving variables in the lipid library. This was followed by SIMPER analysis using the simper function in the vegan package [25]. Further, cluster analyses of the lipid pattern within each group were conducted with the hclust function in the stats package [26], with non-squared euclidian distances being used.

Pairwise tests for group differences were conducted using permutational multivariate analysis of variance using distance matrices (ADONIS) via the adonis function in the vegan package. ADONIS was chosen as it is described as a robust analogue to a parametric MANOVA [27,28]. To adjust for repeated measurement, p-values obtained from ADONIS were Bonferroni-adjusted. Furthermore, multivariate dispersion homogeneity was tested via the betadisper function also supplied by the vegan package.

Leave one prey out analysis (LOPO) was performed using the lopo function in the qfasar package [29]. All zero values (i.e. fatty acids not detected) in the data were scaled to 75% of the smallest value in the dataset to remove non-essential zeros [30], and data was rescaled to sum to 1.0. Aitchison distance was used in the distance measurement.

All statistical analysis were carried out using R version 3.5.0 [26].

## Results

### 3.1 Lipid library

The TLFA patterns of major basal resources in soil food webs were assessed from a total of 50 different species. The tissue analyses of algae, bacteria, fungi and plants resulted in a combined lipid library comprising 62 bacteria, 60 fungi, 59 algae and 48 plant samples, with the latter separated in 24 leaf tissue and 24 root tissue samples (n = 229; S1 Table). In total, 70 fatty acids ranging from 10 to 24 carbon atoms chain length were identified and quantified via GC-FID and GC-MS, and of these, 59 fatty acids where observed with more than 1% relative proportion in at least one sample. However, none of the investigated organisms featured the total range of 70 nor the 59 predominant fatty acids, but rather represented a specific subset of the overall pattern (S3 Table).

Bacteria showed the most diverse lipid patterns, featuring 5 to 25 fatty acids, with many molecules not found in other organism groups such as iso-, anteiso- and cyclic forms (Table 1). Fungi generally comprised low number of fatty acids, ranging from 2 in one sample of *Agrocybe gibberosa*, consisting entirely of 16:0 and 18:2ω6, to *Cladosporium* sp. with 10 fatty acids as most diverse profile. Algal lipid patterns were less diverse than those of bacteria, with 9 to 19 different fatty acids, and 18:1ω9, 18:3ω3,6,9 and 16:3ω3,6,9 as major molecules. Finally, plant lipids comprised 5 to 12 fatty acids, with α-linolenic acid (18:3ω3,6,9) dominating the profile. Overall, the most common fatty acids across groups were 16:0, 18:1ω9, 18:2ω6,9, 18:3ω3,6,9, as well as various methyl-branched iso and anteiso types specific for bacteria (S3 Table).

**Table 1:** Composition of fatty acid profiles.

Organism	N_FA_ and range	Saturated (%)	Mono-unsaturated (%)	Poly-unsaturated (%)	Medium chain (%)	Long chain (%)
**Bacteria**	11.8 (5–25)	73. 9	26.7	0	95.6	4.3
**Fungi**	5.8 (2–10)	23.6	28.7	47.6	23.3	76.7
**Algae**	14.5 (9–19)	15.1	11.0	73.8	35.9	64.0
**Plants** **Leaves**	8.0 (7–11)	20.4	1.2	78.3	22.6	77.4
**Roots**	9.9 (5–12)	35.9	4.8	59.4	31.7	68.3

Mean number of fatty acids (N_FA_), range (min-max) and mean proportion (%) of molecule types (mono-/polyunsaturated, medium-/long chain) across different organism groups representing major soil food web resources. Medium chain– 10 to 18 carbon atoms, long chain– 20 and more carbon atoms.

### 3.2 Distinction in fatty acid signatures at group level

**Fig 1:**
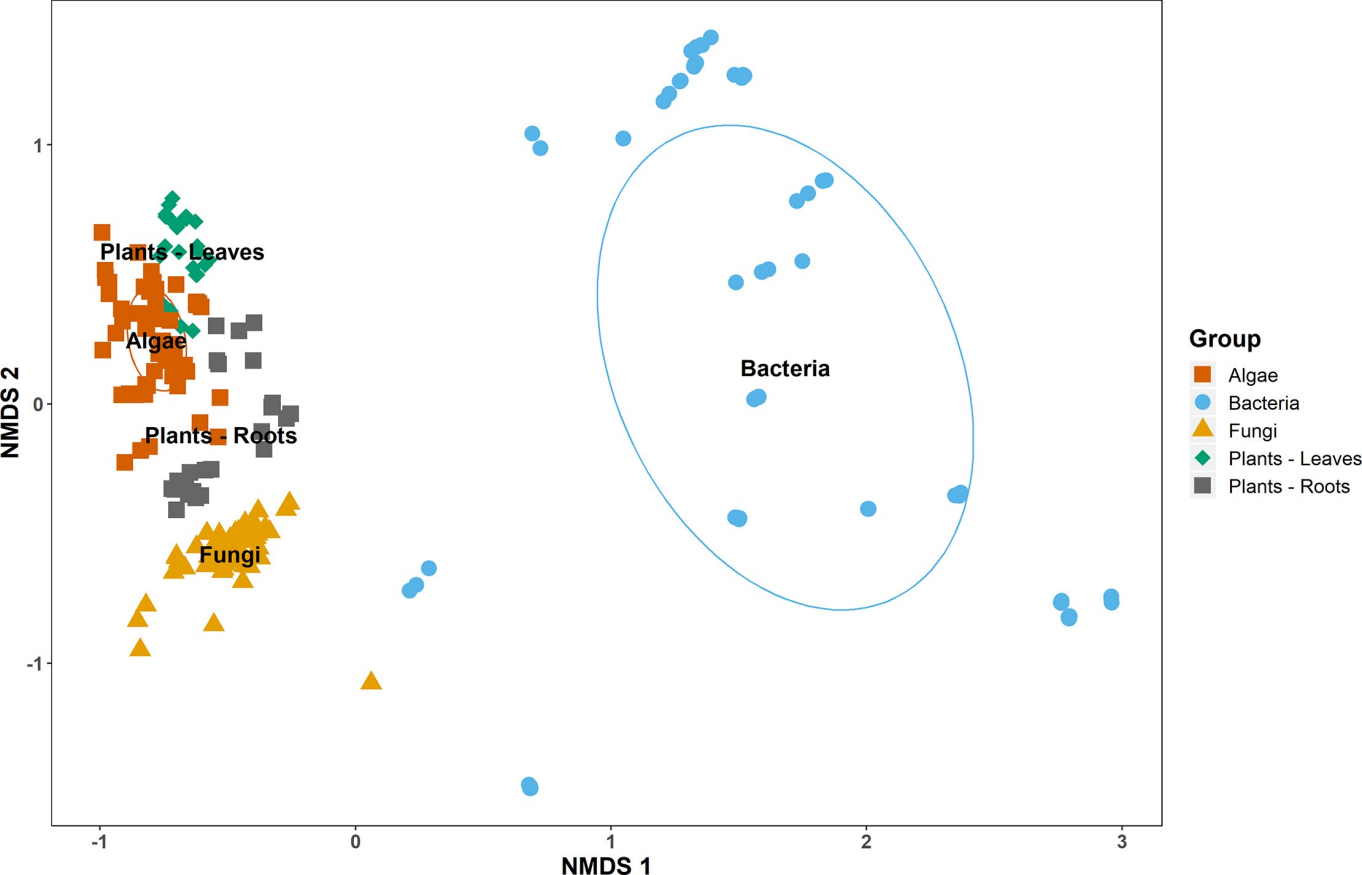
Non-metric multidimensional scaling plot. Results of non-metric multidimensional scaling (nMDS) analysis of the entire lipid library on major soil food web resources using n = 229 samples and m = 59 fatty acids as variables. The overall stress is 0.12. Normal confidence ellipses at α = 0.05.

Multivariate analyses were applied to assess whether the fatty acid composition of plants, algae, fungi and bacteria are sufficiently different for group distinction of food web resources. As a first step, ordination analysis was applied for the visualisation of data distribution. The nMDS separated the different groups, most distinctly the bacteria (Fig 1). These were almost completely separated along the first major axis, with only a handful of samples appearing closer to fungi than to other bacteria. These samples belonged to the bacteria *Micrococcus phlei* and *Nocardioides zeicaulis*. Moreover, the bacteria were distinctly clustered into smaller subgroups indicating the heterogeneity of the sampled clades. The second major axis resulted in a discrimination of the other organism groups (Fig 1). While algae and plant leaves were slightly bulked, plant roots and, most prominent, fungi were separated from each other by their fatty acid pattern.

To scrutinise this picture, in a second step ADONIS was performed to assign statistical difference between the groups separated on the second major axis of the nMDS. Plant roots and fungi were significantly different (F_*84*, *23*_ = 54.94, *P*_*adj*._ *=* 0.004), as well as plant roots and algae (F_*83*, *23*_ = 67.34, *P*_*adj*._ *=* 0.004), and plant leaves and algae (F_*83*, *23*_ = 62.50, *P*_*adj*._ *=* 0.004). Moreover, plant leaves and roots showed varying lipid pattern (F_*48*, *18*_ = 102.78, *P*_*adj*._ *=* 0.004). These findings indicate the distinctiveness of the nMDS groups. However, testing for multivariate dispersion homogeneity resulted in significant differences in variance spread for plant leaves and algae (F_*83*, *23*_ = 15.32, *P <* 0.001) as well as plant leaves and roots (F_*48*, *18*_ = 8.39, *P =* 0.006) data, pointing to caution regarding the discrimination of these groups.

### 3.3 Lipid pattern of common crop plants

**Fig 2:**
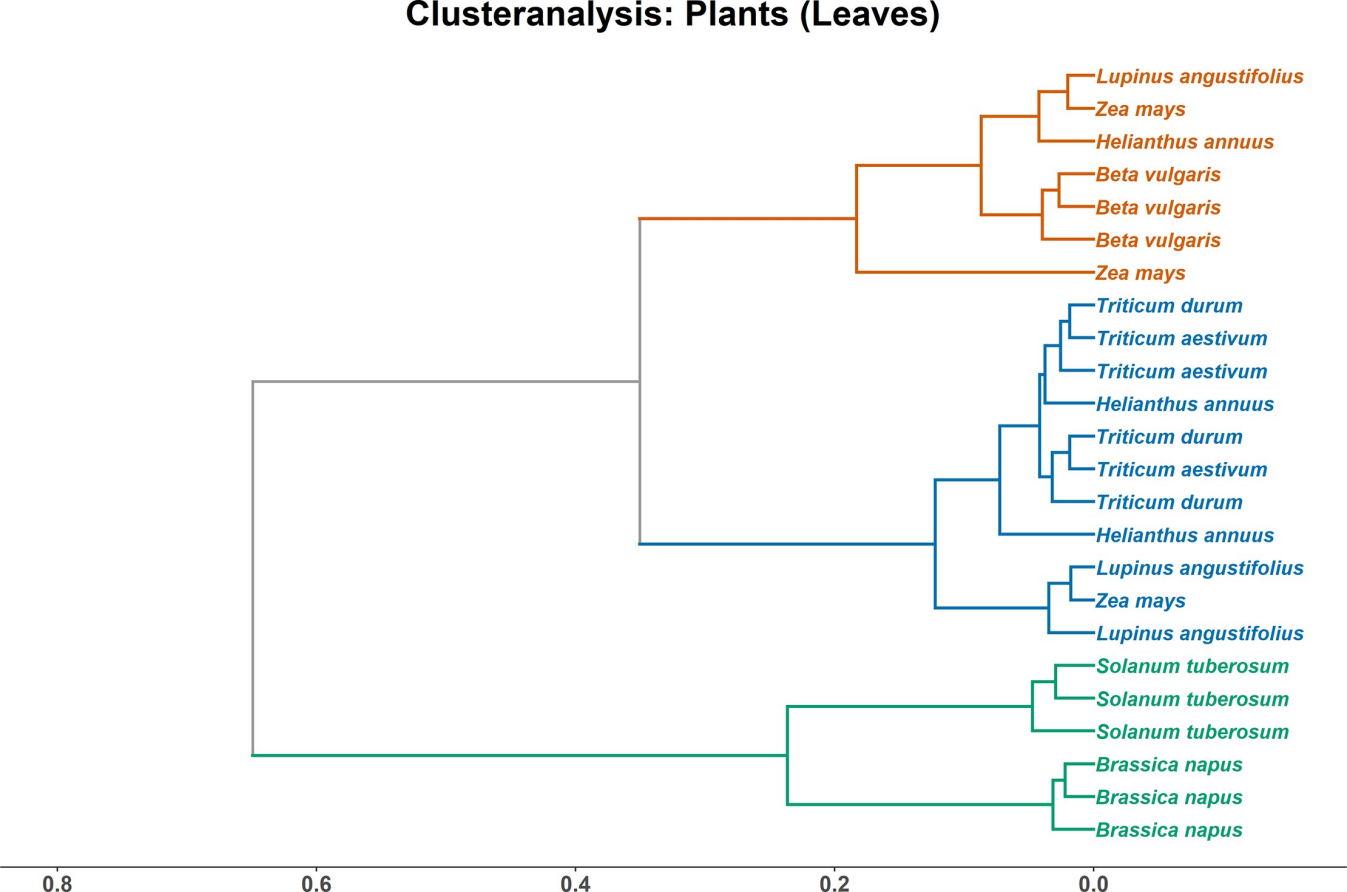
Clusteranalysis of total lipid fatty acids (TLFA) from plants leaves. Axis shows non-squared Euclidean distance.

The eight investigated crop plants originated from a single agricultural site, i.e. grown in the same soil, management practice and climate. To assess if, despite these similar abiotic conditions, plants display a species or group specific lipid pattern, cluster analyses on leaf and root tissue were conducted. In the leaf tissue, the samples of the species *Solanum tuberosum* and *Brassica napus* clustered apart from all other species, but additionally were clearly separated from each other (Fig 2). Both crops exhibited high levels of 16:3ω3,6,9, which was lacking in all other plant leaf samples. A second distinct cluster was formed by wheat, including the samples of both *Triticium aestivum* and *T*. *durum*, with *Helianthus annuus* close by. This cluster featured the highest levels of 18:3ω3,6,9, with up to 70% versus 50 to 60% in the other clusters, but lower levels of 18:2ω6,9. *Lupinus angustifolius* and *Zea mays* were dispersed across several clusters, partly in a third cluster with *Beta vulgaris* and one *Helianthus annuus* sample. This cluster is characterised by the highest proprtion of 16:0 with up to 20%. Overall, while forming distinct clusters, euclidean distance was not very large (Fig 2).

**Fig 3:**
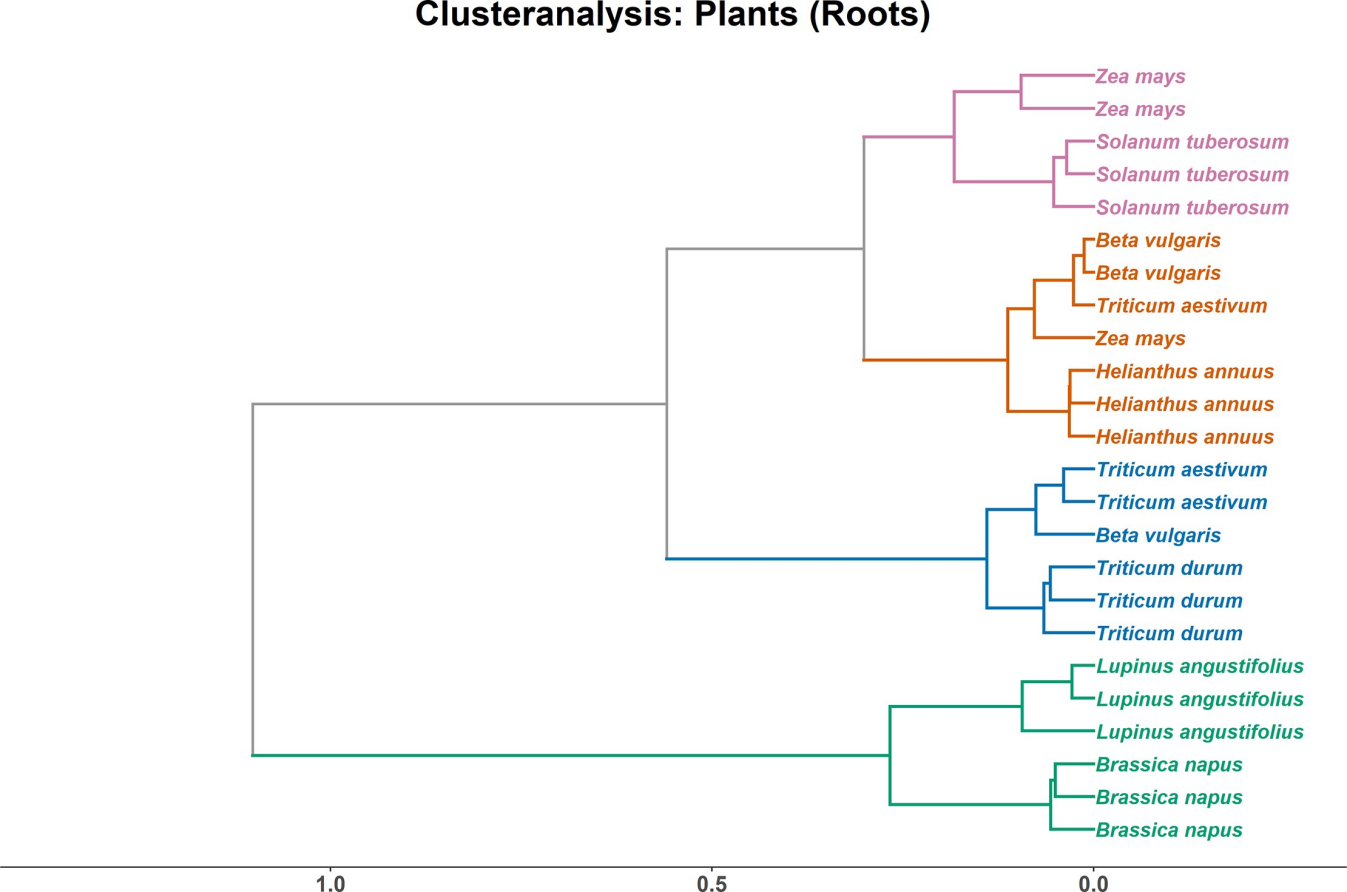
Clusteranalysis of total lipid fatty acids (TLFA) from plants roots. Axis shows non-squared Euclidean distance.

Similar to leaves, the root samples of *Brassica napus* were separated from other crops, here together with *Lupinus angustifolius*. This cluster is characterised by comparably high levels of 18:3ω3,6,9, with 40% twice as common compared to the 10–20% in the other root samples. As a result of this predominance of 18:3ω3,6,9, the proportion of 18:2ω6,9 was much lower than in the other plant roots, where 18:2ω6,9 was the main fatty acid. Three other clusters are mostly separating species as well: Firstly, a cluster made up of *Triticum* samples, characterized by 3% of 16:1ω7, which only appeared in trace amounts in the other plants. Secondly, a *Solanum tuberosum* and *Zea mays* cluster, characterised by the highest proportion of 16:0. Thirdly, an *Helianthus annuus* and *Beta vulgaris* cluster featuring the highest proportion of 18:2ω6,9, with 55% of all fatty acids. Overall, while crops were more distinctly separated by their root lipids compared to leave lipids, Euclidean distance between the clusters is still pretty low (Fig 3).

### 3.4 Library specificity

An important prerequisite to a successful implementation of a quantitative food web model in soil ecosystems is the distinctiveness of the underlying resource/prey library. For this, SIMPER analysis was conducted, to assess the average dissimilarity between organism groups and the main contributing fatty acids. Table 2 presents an overview of the SIMPER results. As already assigned by the nMDS, the biggest dissimilarity was observed between bacteria and all other organisms, as many bacterial fatty acids are exclusive to this group. Moreover, many fatty acids common in the other resources, especially long-chain forms, are hardly detected in bacteria. Averaged over all bacteria, the fatty acid a15:0 is the most influential in separating bacteria from other groups, based on its high abundance in bacterial signatures. In contrast, differences between the other groups tested are smaller, with the lowest dissimilarity between algae and plant leaves as well as between fungi and plant roots. The separation between the groups is a result of the presence, absence or relative levels of the fatty acids 18:3ω3,6,9, 18:2ω6,9, 18:1ω9, 16:3ω3,6,9 and 16:1ω7. Both 18:3ω3,6,9 and 16:3ω3,6,9 are common in plants and algae, whereas high levels of 18.2ω6,9 is typical for fungi.

**Table 2:** SIMPER analysis.

Groups	Average group dis-similarity	Fatty acids	Contributions of FA to group dissimilarity	Average FA dissimilarity	Average FA abundance	
**Bacteria & Fungi**	88.74				**Bacteria**	**Fungi**
		**18:2**ω**6**	26.86	23.83	0.00	47.63
		**a15:0**	14.33	12.71	25.42	0.00
		**18:1**ω**9**	13.61	12.08	3.72	25.69
**Bacteria & Algae**	90.94				**Bacteria**	**Algae**
		**18:3**ω**3**	16.20	14.73	0.00	29.44
		**a15:0**	13.98	12.71	25.42	0.00
		**18:2**ω**6**	10.29	9.36	0.00	18.70
**Bacteria & Plants (L)**	91.64				**Bacteria**	**Plants (L)**
		**18:3**ω**3**	34.23	31.37	0.00	62.71
		**a15:0**	13.87	12.71	25.42	0.00
		**16:1**ω**7**	8.80	8.06	16.33	0.66
**Bacteria & Plants (R)**	89.11				**Bacteria**	**Plants (R)**
		**18:2**ω**6**	21.80	19.43	0.00	38.84
		**a15:0**	14.20	12.65	25.42	0.60
		**18:3**ω**3**	11.52	10.27	0.00	20.53
**Fungi & Algae**	60.14				**Fungi**	**Algae**
		**18:2**ω**6**	24.74	14.88	47.63	18.70
		**18:3**ω**3**	24.49	14.73	0.00	29.44
		**18:1**ω**9**	16.25	9.77	25.69	9.97
**Fungi & Plants (L)**	69.27				**Fungi**	**Plants (L)**
		**18:3**ω**3**	45.34	31.41	0.00	62.71
		**18:2**ω**6**	24.33	17.55	47.63	12.99
		**18:1**ω**9**	18.17	12.58	25.69	0.59
**Fungi & Plants (R)**	41.01				**Fungi**	**Plants (R)**
		**18:1**ω**9**	28.06	11.52	25.69	3.14
		**18:3**ω**3**	25.01	10.27	0.00	20.53
		**18:2**ω**6**	20.23	8.31	47.63	38.84
**Algae & Plants (L)**	42.49				**Algae**	**Plants (L)**
		**18:3**ω**3**	39.29	16.70	29.44	62.71
		**16:3**ω**3**	17.66	7.50	16.98	2.45
		**18:1**ω**9**	11.33	4.81	9.97	0.59
**Algae & Plants (R)**	48.15				**Algae**	**Plants (R)**
		**18:2**ω**6**	22.74	10.95	18.70	38.84
		**16:3**ω**3**	17.64	8.49	16.98	0.00
		**18:3**ω**3**	15.89	7.65	29.44	20.53
**Plants (L) & Plants (R)**	46.30				**Plants (L)**	**Plants (R)**
		**18:3**ω**3**	45.68	21.15	62.71	20.53
		**18:2**ω**6**	28.04	12.98	12.99	38.84
		**16:0**	9.81	4.54	17.94	26.89

Results of the SIMPER Analysis of total lipid fatty acid (TLFA) pattern using the Bray-Curtis Dissimilarity index. L = leaves, R = roots.

Aside from the overall distinctiveness and the primary contributors, it is important to assess the specificity of resources in the context of quantitative modelling. The prior investigation of any cofounding and estimation errors in the lipid library is a key part in ensuring the overall quality of a consumer diet estimation in modelling. Applying the leave one prey out (LOPO) approach resulted in a mean correct estimation of 88%. The correct estimation of diets in the specific resource group as well as their main cofounding error are displayed in Table 3. With the exception of the algae group, all feeding resource groups are near or above 90% correct estimation. However, one in three algae samples has been misidentified as aboveground plant matter in the LOPO analysis, resulting in a potential underrepresentation of algae resource and overrepresentation of plant resource in a consumer diet.

**Table 3:** “Leave-one-prey-out” analysis.

Group	Mean correct [%]	Total correct [%]	
	88.03	84.47	
	**Correct [%]**	**Error–Main**	**Error [%]**
**Algae**	60.04	Plants—Leaves	35.47
**Bacteria**	84.67	Fungi	10.30
**Fungi**	98.08	Plants—Roots	1.65
**Plants—Leaves**	97.80	Fungi	0.91
**Plants—Roots**	88.14	Fungi	6.43

Results of LOPO Analysis on “group” level. Given are the mean and total correct identification for every level, as well as correct identification of each resource type, their main misidentifier (error–main) and its percentage (error %). Used in the analysis were n = 229 samples with F_fa_ = 59 variables, belonging to M_group_ = 5 classifications. Used measurement of distance was the Aitchison distance.

## Discussion

### 4.1 Diversity and specificity of the lipid library

The lipid pattern of the investigated soil food web resources differed in the number of fatty acids and their relative proportions as well as the dominant components and specific marker fatty acids. Most striking was the variation in overall diversity of fatty acid signatures across groups. Particularly bacteria, which differ in lipid metabolisms in comparison to eukaryotic species [31], are highly distinct from the other groups investigated, and were separated by nMDS along the first axis. This is not only apparent by the high number of fatty acids in bacterial tissue, but also by the occurrence of several unique molecules such as cyclic or methyl-branched types. These characteristics are due to the bacteria-specific enzyme complexes branched-chain and straight-chain fatty acid synthetase [32]. Moreover, the investigated bacterial taxa contained no ω3 polyunsaturated fatty acids (PUFAs), typically observed in plants and fungi, which accomplish the desaturation of fatty acids in the methyl-direction of the molecule [33]. As a result, bacterial signatures are strongly group specific, making energy pathways through the bacterial decomposer channel easy to allocate in community or multi-trophic food web investigations.

In contrast, fungi, algae and plants were less well discriminated by nMDS, yet significantly separated by ADONIS. The diversity and specificity of these major resource groups mainly hinges on 5 to 6 fatty acids, with the main indicators being the presence or absence of ω3 PUFAs, distinguishing organism (or tissues) with and without photosynthetic activity. This was clearly visible in the SIMPER analysis, revealing lowest dissimilarity between algae and aboveground (autotroph) plant tissue, whereas belowground (heterotroph) plant tissue was close to fungi.

Across fungi, algae and plants, the majority of fatty acids had a 16 to 18 chain length, constituting up to 95% in algae, and 98% in fungi and leaf tissue. Two relative (18:1ω9, 18:2ω6,9) and two absolute trophic markers (16:3ω3,6,9, 18:3ω3,6,9) create the observed pattern from fungi over roots to leaves and algae as visualised by the distinction along the second axis in the nMDS. It is therefore important to pay attention to these fatty acids in food web analysis, both in qualitative and quantitative studies. Especially ω3 PUFAs have often been underrepresented in previous studies, due to technical constraints such as the frequent co-elution of α-linolenic acid (18:3ω3,6,9) with oleic acid (18:1ω9), when using non-polar columns in gas chromatography. Moreover, the striking difference in major basal food web resources between terrestrial and marine ecosystems has to be taken into account. In marine food webs ω3 PUFAs provided by autotrophic organisms (i.e. planktonic algae) are mainly long-chain forms such as eicosapentaenoic acid (20:5ω3,6,9,12,15) and docosahexaenoic acid (22:6ω3,6,9,12,15,18) [34]. In contrast, terrestrial photoautotrophs deliver C_18_-PUFA (α-linolenic acid by vascular plants) and C_16_-PUFA (16:3ω3,6,9 by micro-algae), which can be used for resource discrimination in food web analyses.

### 4.2 Plant lipids in above- and below-ground tissue

Cluster analyses of leaf and root tissue of crops resulted in clusters of different groups of species, with cultivars partly clustering away from other cultivars of the same crop. In both above- and below-ground plant biomass, a cluster with a large distance towards the others was apparent, always comprising *Brassica napus*, yet joined with *Solanum tuberosum* in leaf tissue and with *Lupinus angustifolius* in root tissue. The fusion in leaf tissue is a direct result of the occurrence of 16:3ω3,6,9 in *B*. *napus* and *S*. *tuberosum*, a ω3 PUFA, which is lacking in all other investigated crops. This is an indication of the plastidial fatty acid biosynthesis pathway encountered in algae and some plants, including *B*. *napus* and *S*. *tuberosum*, while in most other plants this metabolism was lost during evolution and only an extra-plastidial pathway remained [35].

In contrast, this first distinct cluster in root tissue formed by *B*. *napus* and *L*. *angustifolius* originates from low levels of linoleic acid and high levels of α-linolenic acid. Both plants are non-mycorrhizal [36], although *L*. *angustifolius* was shown to be colonised by fungal symbionts, albeit very poorly [37]. However, the common marker for arbuscular mycorrhiza fungi (16:1ω5) [38] was not detected in any of the root tissues sampled, reflecting the fact that mycorrhization of crop plants generally is very low due to regular fertilization. The change in the ratio of α-linolenic to linoleic acid likely indicates altered rhizosphere communities in *B*. *napus* and *L*. *angustifolius*. Linoleic acid is widespread in fungi [39,40,6] and commonly applied as a marker for soil fungi [41]. Particular Ascomycetes and Basidiomycetes contain 18:2ω6,9, with 36 to 61% and 45 to 57%, respectively, of the total phospholipid fatty acids [42]. The low levels of linoleic acid therefore indicate that, besides the lack of arbuscular mycorrhiza, also saprotrophic taxa have a low abundance in the rhizosphere of *B*. *napus* and *L*. *angustifolius*.

Clustering of the other crops was less clearly linked to the presence or absence of a specific fatty acid, but rather a result of variations in proportion of major fatty acid markers related to differences in lipid metabolism. Again, α-linolenic acid and linoleic acid were important for the fusion of the two *Triticum* clusters in leaves, whereas for roots it was palmitoleic acid (16:1ω7). Generally, linoleic acid forms the precursor for α-linolenic acid production in plants [31, 33], and this pathway is important for the overall PUFA level and in turn the maintenance of adequate viscosity and selective permeability of membranes [43]. Varying ratios of these two fatty acids might reflect adaptations of the different crops to environmental conditions, e.g. PUFA levels can be linked to temperature tolerance [44].

In leaf tissue, three clusters were apparent, whereas roots separated in four clusters. The species clustering together were different for leaf and root tissue, with roots showing a better alignment. In roots, 5 out of the 8 crops joint in a single cluster, showing a good species-specific distinction independent of cultivar. The remaining three crops, *Beta vulgaris*, *Triticum aestivum* and *Zea mays*, also grouped together, yet with one outlier each. As the fatty acid signatures of root tissue not only incorporate the physiological differences of the plant metabolism, but also the rhizosphere communities, roots likely exhibit a greater fatty acid diversity and separation potential. The rhizosphere community of plants is predominantly shaped by plant species and soil type [45] and to a lesser degree by cultivar [46]. In the present study, soil type and agricultural management were the same across crops. This implies that, besides plant species, the specific rhizosphere community (and the fatty acids patter derived thereof) considerably contributed to the clustering of crop species, whereas crop cultivar was less important.

### 4.3 Applicability of the lipid library for quantitative modelling

The constructed lipid library is a prerequisite to perform quantitative modelling in food web analyses and most important here are the distinctiveness and specificity of resources. The lipid library established by the present study was divided into four groups (plant, algae, bacteria, fungi), representing the major resources of the herbivore and detrital food chain. Based on LOPO analyses the distinctiveness of groups was very good, approaching 90%, with a mean correct estimation of 88%. However, while most groups were correctly identified and estimation was as high as 98% in fungi, algae reached only 60%, with a 35% cofounding error confusing algae samples for plant leaf tissue.

As stated above, a portion of the investigated plants use a plastidial pathway for fatty acid synthesis [35], and thus comprise 16:3ω3,6,9, also common in algae. In fact, the 25% of leaf tissue samples featuring this fatty acid match the 35% cofounding error, and an investigative cluster analysis using both algae and leaf data show that leaves containing 16:3ω3,6,9 cluster with 17 of the 59 algae samples (S1 Fig). Interestingly, no cofounding takes place in the leaf data set. Therefore, researchers must pay attention to the reported levels of algae and aboveground plant matter in a consumers diet, as there might be an overestimation of plant diet and an underestimation of algae diet. As this bias is mainly due to the occurrence of 16:3ω3,6,9 in the resource, it might be an option to exclude that fatty acid from the library, but this is only appropriate if overall correct estimation by LOPO does not suffer from it. Considering that 16:3ω3,6,9 is an absolute marker fatty acid for algal feeding [9], removal likely will hamper determination of food chains based on, e.g. biological soil crusts.

While LOPO analyses indicated a high quality of the library, this doesn’t necessarily inform about the ability of a quantitative model to accurately estimate predator diets. The performance of a QFASA model depends on many factors, apart from the prey library is the specific diet of a consumers, the accuracy of calibration coefficients [47], the chosen distance measurement [48] and the correct handling of the underlying dataset [49]. Nonetheless, a lipid library that with around 90% accuracy performs correct estimates of group (resource) membership of every included sample, represents a promising data set and warrants further exploration in soil food web research.

## Supporting information

S1 Fig Clusteranalysis of total lipid fatty acids (TLFA) from algae and plant roots. Clusteranalysis highlighting the nesting of two plant species (*S*. *tuberosum* and *B*. *napus*) within the algae cluster. Axis shows non-squared Euclidean distance.
(TIFF)

S1 Table Data overview. Overview on taxa and sample numbers used to construct a lipid library comprising the four major basal food web resources in soil, plants, algae, fungi, and bacteria.
(DOCX)

S2 Table Agronomic management. The agronomic management at the log-term field trial, where the leave and root samples of the different crops were taken on the 8^th^ of June 2017. Mineral fertilisation with nitrogen (N), phosphorus (P) and potassium (K) (kg ha^−1^) and chemical plant protection, time of application and name of the pesticide applied (in parentheses) during the cropping period. This management practice has been constant since 2006.
(DOCX)

S3 Table Lipid library. Total lipid fatty acids (TLFA) in tissues of plants, algae and bacteria. Overall 62 bacteria, 59 algae and 48 plant samples were investigated, with the latter separated in 24 leaf and 24 root tissue samples. Fungal data used in the analyses can be found in Ruess et al. 2002 [21] and Ruess et al. 2004 [10].
(XLSX)
