# Supplementary figures and images for: Diversity and specificity of lipid patterns in basal soil food web resources
Source: PLoS One. 2019 Aug 20;14(8):e0221102. doi: 10.1371/journal.pone.0221102 (PMC6701827; doi:10.1371/journal.pone.0221102)

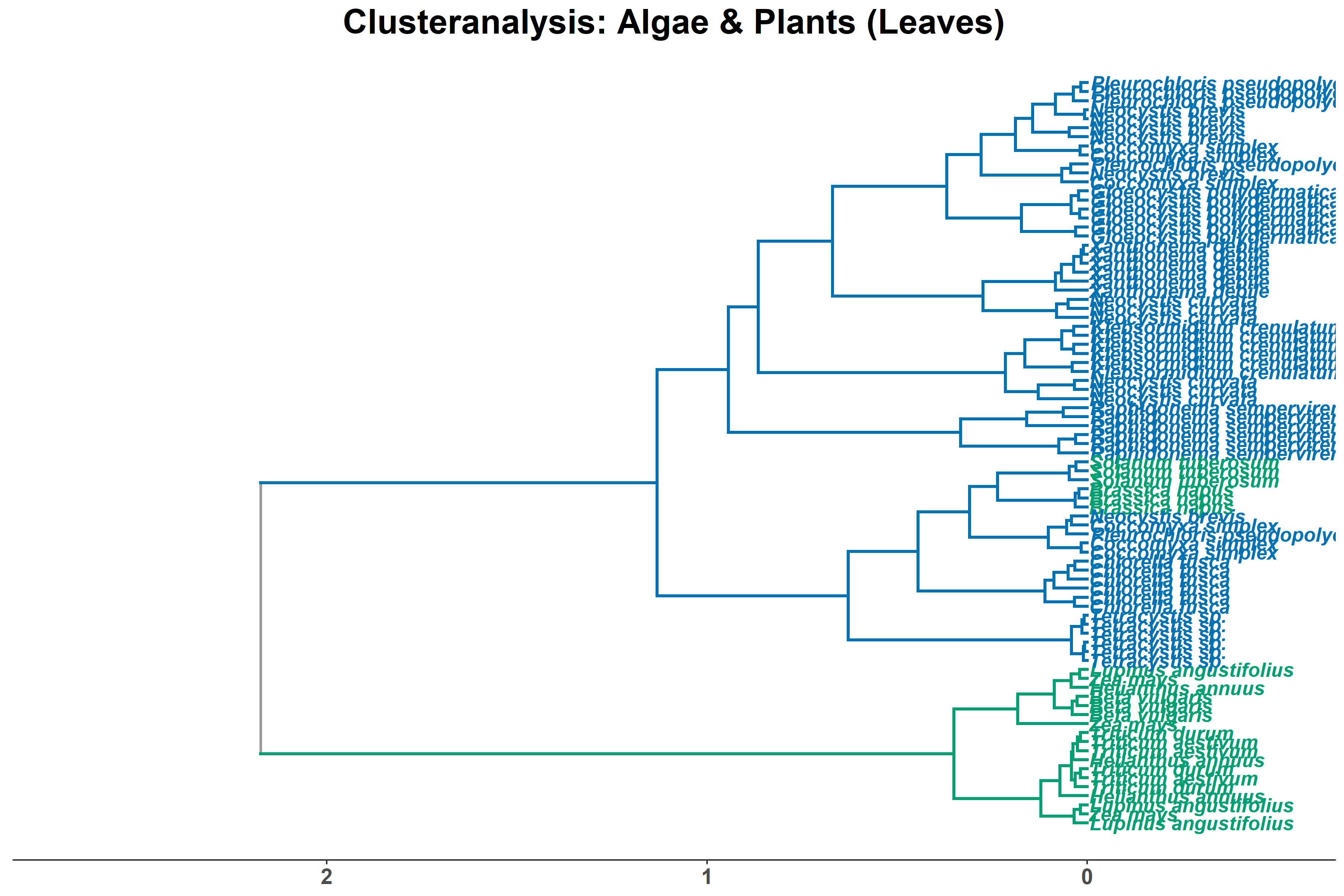

Supplement: S1 Fig — Clusteranalysis highlighting the nesting of two plant species (S. tuberosum and B. napus) within the algae cluster. Axis shows non-squared Euclidean distance. (TIFF) [file pone.0221102.s001.tiff]
