# Supplementary material for: Diversity and specificity of lipid patterns in basal soil food web resources
Source: PLoS One. 2019 Aug 20;14(8):e0221102. doi: 10.1371/journal.pone.0221102 (PMC6701827; doi:10.1371/journal.pone.0221102)
Supplement: S1 Table — Overview on taxa and sample numbers used to construct a lipid library comprising the four major basal food web resources in soil, plants, algae, fungi, and bacteria. (DOCX) [file pone.0221102.s002.docx]

**S1 Table.** **Data Overview.**

| Plants | Algae | Fungi | Bacteria |
| --- | --- | --- | --- |
| *Beta vulgaris*  (Leaf n = 3, root n = 3) | *Chlorella fusca* (n = 6) | *Agrocybe gibberosa* (n = 7) | *Agromyces cerinus* (n = 4) |
| var. Annemaria  var. Beretta  var. Hannibal | *Coccomyxa simplex* (n = 6) | *Alternaria* sp. (n = 3) | *Agromyces terreus* (n = 4) |
| *Brassica napus*  (Leaf n = 3, root n = 3) | *Gloeocystis polydermatica*  (n = 6) | *Cenococcum graniforme*  (n = 2) | *Arthobacter siccitolerans* (n = 3) |
| var. Heros  var. Campino  var. Belinda | *Klebsormidium crenulatum* (n = 6) | *Chaetonium globosum*  (n = 7) | *Bacillus megaterium* (n = 4) |
| *Helianthus annuus*  (Leaf n = 3, root n=3) | *Neocystis brevis* (n = 6) | *Chrysosporium* sp. (n = 3) | *Bacillus subtilis* (n = 4) |
| var. Bella  var. Delfi  var. PR 65 H 22 | *Neocystis curvata* (n = 6) | *Cladosporium* sp. (n = 3) | *Cellulomas flavigens* (n = 4) |
| *Lupinus angustifolius* (Leaf n = 3, root n = 3) | *Pleurochloris pseudeopolychloris* (n = 6) | *Curvularia* sp*.* (n = 3) | *Curtobacterium herbarum* (n = 4) |
| var. Mirabor  var. Haags Blaue  var. Boruta | *Raphidonema sempervirens* (n = 5) | *Epicoccum sp.* (n = 3) | *Edaphobacter lichenicola* – A2108 M8UP22 (n = 4) |
| *Solanum tuberosum* (Leaf n = 3, root n = 3) | *Tetracystis sp.* (n = 6) | *Hymenoscyphus ericae*  (n = 3) | *Edaphobacter lichenicola* – A2109 M8UP30 (n = 4) |
| var. Jelly  var. Gala  var. Finka | *Xanthonema debile* (n = 6) | *Laccaria laccata* (n = 6) | *Granulicella mallensis* - A528 MP5 ACTX8 (n = 4) |
| *Triticum aestivum*  (Leaf n = 3, root n = 3) |  | *Monocilium* sp*.* (n = 2) | *Granilicella mallensis* – B2272 XP53 (n = 4) |
| var. Heliaro  var. KWS Chamsin  var. Sagittario |  | *Paxillus involutus* (n = 2) | *Micrococcus luteus* (n = 4) |
| *Triticum durum*  (Leaf n = 3, root n = 3) |  | *Penicillium sp.* 1 (n = 3) | *Micrococcus phlei* (n = 4) |
| var. Duramonte  var. Floradur  var. Lupidur |  | *Penicillium* sp*.* 2 (n = 3) | *Nocardioides zeicaulis* (n = 4) |
| *Zea mays*  (Leaf n = 3, root n = 3) |  | *Phoma* sp*.* (n = 3) | *Streptomyces cacoi* (n = 4) |
| var. Sucorn  var. Perrero  var. Amazing |  | *Saccharomyces cerevisiae*  (n = 2) | *Terriglobus saanensis* (n = 4) |
|  |  | Species steti 1 (n = 3) |  |
|  |  | Species steti 2 (n = 2) |  |
| SUM: 48 | **SUM: 59** | **SUM: 60** | **SUM: 62** |

Overview on taxa and sample numbers used to construct a lipid library comprising the four major basal food web resources in soil, plants, algae, fungi, and bacteria.
