# Supplementary material for: Diversity and specificity of lipid patterns in basal soil food web resources
Source: PLoS One. 2019 Aug 20;14(8):e0221102. doi: 10.1371/journal.pone.0221102 (PMC6701827; doi:10.1371/journal.pone.0221102)
Supplement: S2 Table — The agronomic management at the log-term field trial, where the leave and root samples of the different crops were taken on the 8th of June 2017. Mineral fertilisation with nitrogen (N), phosphorus (P) and potassium (K) (kg ha−1) and chemical plant protection, time of application and name of the pesticide applied (in parentheses) during the cropping period. This management practice has been constant since 2006. (DOCX) [file pone.0221102.s003.docx]

**S2 Table. Agronomic Management.**

| Crop | N | P | K | Chemical plant protection |
| --- | --- | --- | --- | --- |
| *Brassica napus* | 150 | 26 | 116 | - |
| *Triticum aestivum* | 60 | 0 | 100 | Herbicide 12^th^ May (Biathlon, Starane XL)  Fungicide 22^nd^ May (Gladio) |
| *Triticum durum* | 60 | 0 | 100 | Herbicide 12^th^ May (Biathlon, Starane XL)  Fungicide 22^nd^ May (Gladio) |
| *Lupinus angustifolius* | 20 | 0 | 100 | Herbicide 31^st^ March (Stomp Aqua, Boxer) |
| *Helianthus annuus* | 60 | 0 | 100 | - |
| *Solanum tuberosum* | 90 | 26 | 166 | Herbicide 18^th^ May (Sencor Liquid)  Fungicide 14^th^ June (Acrobat Plus WG)  Fungicide 3^rd^ July (Ridomil Gold MZ)  Insecticide 14^th^ June, 3^rd^ July (Biscaya) |
| *Beta vulgaris* | 120 | 26 | 166 | Herbicide 11^th^ & 24^th^ May, 8^th^ June (Betanal Expert, Golfix Gold, Oleo FC) |
| *Zea mays* | 90 | 26 | 133 | Herbicide 1^st^ June (Spectrum Gold, Maran, Kelvin OD) |

The agronomic management at the log-term field trial, where the leave and root samples of the different crops were taken on the 8^th^ of June 2017. Mineral fertilisation with nitrogen (N), phosphorus (P) and potassium (K) (kg ha^-1^) and chemical plant protection, time of application and name of the pesticide applied (in parentheses) during the cropping period. This management practice has been constant since 2006.
